# The Hospital World

**Published:** 1910-03-26

**Authors:** 


### The Hospital World.

### Personalia.

Mr. Charles Lupton has been re-elected hon. treasurer of the Leeds Infirmary.

Lord Alverstone has been re-elected president of the London Temperance Hospital.

Viscount Newport has been appointed President of the General Hospital, Birmingham.

Archdeacon Stanhope has been re-elected President of the Hereford Victoria Eye and Ear Hospital.

Prince Alexander of Teck has been re-elected president of the Royal Sea Bathing Hospital, Margate.

Colonel R. H. Rawson, M.P., has been appointed president of the Horley and District Cottage Hospital.

Lord Lichfield has been re-elected president, and Sir T. A. Salt treasurer, of the Staffordshire General Infirmary.

The Duke of Bedford has been elected president, and Mr. Henry Lucas treasurer, of University College Hospital.

The Marquis of Hertford has been re-elected President of the Birmingham and Midland Ear and Throat Hospital.

Squire Crawley has been re-elected president of the Luton Bute Hospital, and Mr. W. R. Phillips vice-president.

Mr. John Tate and Mr. R. M. Young have been re-elected honorary treasurer and honorary secretary to the Royal Victoria Hospital, Belfast.

Alderman O'Keeffe has been re-elected Chairman of the North Staffordshire Joint Small-pox Hospital Board, and alderman Cartlidge Vice-Chairman.

Mr. Williams, who has been secretary of the Luton Bute Hospital for seventeen years, said at the annual meeting he would continue to do his best till the day when a change was felt desirable.

An illuminated address, signed by all the members of the medical staff of the East Suffolk and Ipswich Hospital, has been presented to Mr. P. E. Ripley, in recognition of his services as a member of the board of management in special reference to his share in the scheme for the reconstruction of the hospital buildings.

